# An Extremely Rare Case of Immune Checkpoint Inhibitors–Related Hypoparathyroidism and a Critical Literature Review

**DOI:** 10.1210/jcemcr/luaf097

**Published:** 2025-05-12

**Authors:** Spyridon Kazanas, Amalia Anastasopoulou, Dimitrios Ziogas, Helen Gogas, Anna Angelousi

**Affiliations:** First Department of Internal Medicine, Laiko General Hospital, National and Kapodistrian University of Athens, School of Medicine, Athens 11527, Greece; First Department of Internal Medicine, Laiko General Hospital, National and Kapodistrian University of Athens, School of Medicine, Athens 11527, Greece; First Department of Internal Medicine, Laiko General Hospital, National and Kapodistrian University of Athens, School of Medicine, Athens 11527, Greece; First Department of Internal Medicine, Laiko General Hospital, National and Kapodistrian University of Athens, School of Medicine, Athens 11527, Greece; First Department of Internal Medicine, Unit of Endocrinology, Laiko General Hospital, National and Kapodistrian University of Athens, School of Medicine, Athens 11527, Greece

**Keywords:** hypoparathyroidism, hypocalcemia, immunotherapy, immune checkpoint inhibitors, melanoma

## Abstract

Immune checkpoint inhibitors (ICIs) have been approved to treat a variety of malignancies, including melanoma, improving the prognosis of these patients. However, they also cause a wide spectrum of rare immune-related adverse events (irAEs), not well-described yet. Although many endocrinopathies have been recognized as irAEs, primary hypoparathyroidism has rarely been reported, and thus clinical suspicion remains low. Herein, we describe the case of a 68-year-old female patient with metastatic melanoma who was admitted to the emergency department with acute symptomatic hypocalcemia due to immune-related (ir)-hypoparathyroidism after 2 cycles of nivolumab/ipilimumab. The patient was treated symptomatically, and her calcium levels were normalized. Parathyroid hormone levels were partially restored during the 6 months of follow-up. A literature review was conducted, summarizing all other subjects who developed ir-hypoparathyroidism after exposure to ICI-based regimen. The review identified 10 additional cases of hypoparathyroidism during immunotherapy. Interestingly, all melanoma cases with ir-hypoparathyroidism had received nivolumab/ipilimumab; 3 of them were also screened and detected with positive calcium-sensing receptor (CaSR) antibodies. Primary hypoparathyroidism may acutely manifest with symptomatic hypocalcemia and care providers should be aware of this rare irAE.

## Introduction

Immune checkpoint inhibitors (ICIs) target either T-cell surface molecules, such as programmed cell death protein 1 (PD-1) and cytotoxic T-lymphocyte-associated protein 4 (CTLA-4), or malignant cells surface molecules, such as PD-1 ligand (PD-L1), and induce an immune response against neoplastic cells [1]. Several ICIs have gained approvals in different cancer types and therapeutic settings, with remarkable survival outcomes [2]. Particularly for advanced melanoma, the combination of nivolumab (an anti-PD-1) and ipilimumab (an anti-CTLA-4) reported recently its final long-term survival benefit compared to monotherapies of nivolumab and ipilimumab [3]. However, due to the unrestrained response of activated T-cells even against healthy tissues, ICIs may also cause various immune-related adverse events (irAEs) [4]. The severity of irAEs can vary from mild to potentially life-threatening and any organ or system can be affected [5]. Among endocrine irAEs, thyroid disorders and hypophysitis are the most frequent toxicities, whereas other endocrinopathies, such as primary adrenal insufficiency, central diabetes insipidus and hypoparathyroidism, are only scarcely reported in the literature [6, 7]. As the use of ICIs increases, the spectrum of described irAEs may broaden to include even more rare conditions.

Herein, we present a female patient with metastatic melanoma who developed acute symptomatic hypocalcemia due to the development of primary hypoparathyroidism shortly after the initiation of combination therapy with nivolumab and ipilimumab. We also reviewed the recorded occurrence of ICI-induced hypoparathyroidism in the literature to clarify the diagnostic and therapeutic approach of this rare clinical entity [8-17].

## Case Presentation

A 65-year-old female patient with a medical history of hypothyroidism was diagnosed with stage IA melanoma (T1aN0M0) of the scalp and treated surgically with no further adjuvant treatment according to the established guidelines. Five years later, she presented with multiple liver lesions. A liver biopsy confirmed the metastatic spread of her melanoma to the liver. The patient started a combination treatment of nivolumab (1 mg/kg)/ipilimumab (3 mg/kg) every 21 days. A week after the administration of the second dose of nivolumab/ipilimumab, she was evaluated at the emergency department for progressively worsening perioral numbness and paresthesia in the hands and feet extremities and abdominal cramping. Upon administration of the second cycle, the patient had normal serum levels of thyroid stimulating hormone (TSH) at 1.5 μIU/mL, (reference range: 0.5-4.5 μIU/mL), total triiodothyronine (T3) at 115 ng/dL (1.76 nmol/L) (reference range: 60-180 ng/dL; 0.9-2.8 nmol/L) and free thyroxine (T4) at 1.4 ng/dL (18 pmol/L) (reference range: 0.7-1.9 ng/dL; 9-24 pmol/L) as well as normal serum concentrations of calcium (9.2 mg/dL, reference range: 8.5-10.5; 2.2-2.7 mmol/L) and phosphorus of 3.9 mg/dL (1.26 mmol/L) (reference range: 3.4-4.5 mg/dL, 1.12-1.45 mmol/L). Moreover, the patient reported neither surgery nor radiotherapy to the neck area.

## Diagnostic Assessment

At presentation, the physical examination as well as the computerized tomography of the brain were unremarkable. Blood tests revealed severe hypocalcemia with a serum-corrected calcium of 6.3 mg/dL (1.57 mmol/L) (reference range: 8.5-10.5 mg/dL; 2.2-2.7 mmol/L), increased phosphorus 5.2 mg/dL (1.68 mmol/L) (reference range: 3.4-4.5 mg/dL; 1.12-1.45 mmol/L), normal magnesium 1.9 mg/dL (0.88 mmol/L) (reference range: 1.7-2.2 mg/dL; 0.85-1.10 mmol/L) and low 25-hydroxyvitamin D at 16.5 ng/mL (reference range: 20-40 ng/mL). The electrocardiogram revealed no abnormal findings. Intact parathyroid hormone (PTH) was measured at an inappropriately low value of 8 pg/mL (0.85 pmol/L) (reference range: 14-65 pg/mL; 1.49-6.89 pmol/L), considering the low calcium and 25-hydroxyvitamin D levels. Renal function was intact, with creatinine levels at 0.6 mg/dL (53.04 μmol/L) (reference range: 0.5-1.1 mg/dL; 44-97 μmol/L), urea at 21 mg/dL (3.5 mmol/L) (reference range: 21-43 mg/dL; 3.5-7.2 mmol/L) and an eGFR of 100 mL/min/1.73m^2^. Liver function tests were within normal limits. Serum cortisol and thyroid hormone levels were also normal. The patient was hospitalized for treatment of acute symptomatic hypocalcemia. The diagnosis of a primary immune-related (ir) hypoparathyroidism was retained. Anti-PTH antibodies measured by indirect fluorescence immunoassay (IFA) were found negative, but their analysis was performed only a few days after the completion of steroids for immune-related colitis.

## Treatment

Treatment with intravenous calcium gluconate (4 g daily) was initiated for 3 days, followed by oral substitution with calcium carbonate (3 g daily), active vitamin D (alfacalcidol 1 μg daily) and vitamin D3 (25 000 IU weekly). After 5 days of hospitalization, the patient's symptoms gradually improved and she was discharged with the aforementioned treatment.

## Outcome and Follow-Up

**Figure 1.**
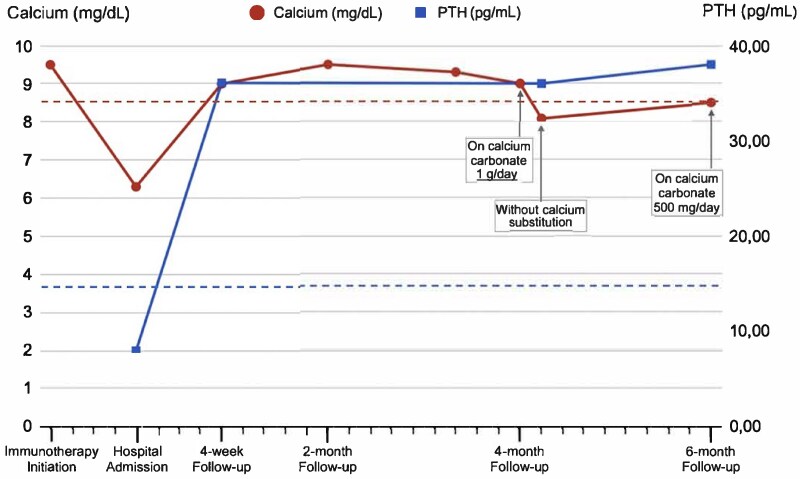
The progression of albumin-adjusted calcium and parathyroid hormone (PTH) levels at diagnosis and during the follow-up period.

Upon the 4-week follow-up visit, the patient was completely asymptomatic, on gradually decreasing doses of calcium carbonate and serum-corrected calcium and PTH levels were within normal values, at 9 mg/dL and 36.1 pg/mL, respectively. After the third cycle of nivolumab/ipilimumab, the patient reported symptoms relevant to ir-colitis grade II treated with prednisolone at a dose of 0.5 mg/kg/day with a 4-week tapering. Four months after the initial diagnosis of ir-hypoparathyroidism, the patient remained asymptomatic on a calcium supplementation dose of 1 g/day, vitamin D3 (25 000 IU weekly), and 0.5 μg alfacalcidol maintaining normal calcium, 25-hydroxyvitamin D and PTH levels motivating the complete interruption of calcium and alfacalcidol substitution. However, a few days after this interruption, calcium levels dropped from 9.0 to 8.1 mg/dL (with normal 25-hydroxyvitamin D at 40 ng/mL) with inappropriate stable levels of the PTH, suggesting a partial recovery of parathyroid function, necessitating thus the maintenance of a low substitution calcium dose of 500 mg/day. At the last follow-up visit, 6 months after the diagnosis, the patient's PTH levels remained stable, and calcium blood levels ranged from 8.5 to 8.8 mg/dL, with normal calcium urine levels (200 mg/24 hours). The progression of serum-corrected calcium and PTH levels is presented in Fig. 1.

## Discussion

Primary hypoparathyroidism is most commonly iatrogenic. Surgical intervention to the anterior neck is responsible for 75% of cases, with the remainder occurring secondary to autoimmune, genetic, or infiltrative etiologies [18]. Autoimmune hypoparathyroidism is the most frequent form of non-iatrogenic hypoparathyroidism in adults, and it can either be isolated or part of autoimmune polyglandular syndrome (APS)-1 [19]. Although rare, autoimmune hypoparathyroidism can also be developed following the use of ICIs [8-17]. The identification of autoimmune hypoparathyroidism is a diagnostic challenge mainly based on clinical criteria of low PTH levels along with hypocalcemia and hyperphosphatemia as were described in all reported cases, including ours [20].

Literature research through the PubMed database identified 10 other cases using the combinations of the following terms: “*hypoparathyroidism*,” “*ICI-induced hypoparathyroidism*,” “*nivolumab*,” “*pembrolizumab*,” “*ipilimumab*,” “*immune-related adverse event*,” and “*immune checkpoint inhibitor*” [8-17]. The main characteristics are summarized in Table 1 [8-17]. Patients’ age ranged between 52 and 76 years. The vast majority of the cases were male (9 cases). Five patients had received immunotherapy due to metastatic melanoma treated with the nivolumab/ipilimumab combination, 5 due to non-small cell lung cancer (NSCLC) (3 were treated with anti-PD-1 monotherapy, 1 with anti-PD-L1 monotherapy, and 1 with anti-CTLA-4 monotherapy), and 1 due to small cell lung cancer (SCLC) (treated with anti-PD-1 monotherapy). All patients presented with severe hypocalcemia and required hospitalization. The time from ICI initiation to the onset of hypoparathyroidism ranged from 28 days to 17 months. Among 9 cases with available data and with a median follow-up of 7 months post-diagnosis, no recovery of hypoparathyroidism was reported. Autoantibody measurement was performed in 6 out of 10 patients. More specifically, calcium-sensing receptor (CaSR) antibodies were searched in 4 patients and found to be positive in 3 of them [11, 13, 15] whereas in the fourth patient, CaSR antibodies were in very low concentration and were also present in controls and thus considered as nonconclusive [12]. In addition to CaSR antibodies, NALP5 antibodies and antibodies against cytokines were also measured in 2 patients [11, 13] and found to be negative. Anti-parathyroid antibodies were measured in one case and were also found to be negative [17], similar to our patient. No other concomitant autoimmune diseases along with hypoparathyroidism have been described in any of the reported cases.

**Table 1.** Summary of cases with ICI-related hypoparathyroidism reported in the literature

Authors	Year	Age	Sex	ICI regimen (Dosing)	Onset after ICI initiation	Neoplasia	Ca levels, (reference range)	PTH levels, (reference range)	Autoantibodies tested, (assay)	Other endocrinopathy	Recovery	Follow-up
Horinouchi et al [8]	2015	ND	ND	Ipilimumab 10 mg/kg (in combination with paclitaxel 175 mg/m^2^ and carboplatin 6 AUC)—phase I	ND	NSCLC	ND	ND	ND	ND	ND	ND
Win et al [9]	2017	73	Male	Ipilimumab (3 mg/kg), Nivolumab (1 mg/kg)	45 days	Melanoma	5.0 mg/dL, 1.24 mmol/L, (ND)	Undetectable (9-80 pg/mL, 0.9-8.9 pmol/L)	ND	Thyroiditis	No	120 days
Umeguchi et al [10]	2018	64	Male	Pembrolizumab 200 mg/Q3W (second line)	60 days	NSCLC	6.5 mg/dL, 1.62 mmol/L, (ND)	8 pg/mL (10-65 pg/mL), 0.84 pmol/L (1-6.8 pmol/L)	ND	No	No	120 days
Piranavan et al [11]	2019	61	Female	Nivolumab (480 mg/month)	240 days	SCLC	5.8 mg/dL (8.3-10 mg/dL), 1.44 mmol/L (2-2.5 mmol/L)	7.8 pg/mL (12-65 pg/mL), 0.82 pmol/L (1.2-6.8 pmol/L)	CaSR Abs(+), (immunoprecipitation assay), NALP5 Abs(−) (Radioligand binding assays), Cytokine Abs(−), (ELISA)	No	No	270 days
Trinh et al [12]	2019	53	Male	Ipilimumab (3 mg/kg), Nivolumab (1 mg/kg)	28 days	Melanoma	5.4 mg/dL (8.4-10.6 mg/dL), 1.34 mmol/L, (2.1-2.65 mmol/L)	7.2 pg/mL (15-65 pg/mL), 0.76 pmol/L (1.5-6.8 pmol/L)	CaSR Abs(detectable but very low), (ND)	No	No	146 days
Dadu et al [13]	2020	73	Male	Ipilimumab (3 mg/kg), Nivolumab (1 mg/kg)	28 days	Melanoma	5.0 mg/dL (8.4-10.2 mg/dL), 1.24 mmol/L (2.1-2.5 mmol/L)	Undetectable (15-65 pg/mL, 1.5-6.8 pmol/L)	CaSR Abs(+), (immunoprecipitation assays, ELISA, and cell-based functional assays), NALP5 Abs(−), (radioligand binding assays), Cytokine Abs(−), (ELISA)	Thyroiditis	No	1185 days
El Kawkgi et al [14]	2020	76	Male	Ipilimumab (3 mg/kg), Nivolumab (1 mg/kg)	210 days	Metastatic Melanoma	6.5 mg/dL (8.8-10.2 mg/dL), 1.62 mmol/L (2.2-2.5 mmol/L)	Undetectable (15-65 pg/mL, 1.5-6.8 pmol/L)	Anti-PTH Abs(−), ND	Hypophysitis	No	77 days
Lupi et al [15]	2020	52	Male	Pembrolizumab 200 mg/Q3W	510 days	NSCLC	6.2 mg/dL (8.6-10.2 mg/dL), 1.54 mmol/L (2.1-2.5 mmol/L)	18 pg/mL (8-40 pg/mL), 1.9 pmol/L (0.8-4 pmol/L)	CaSR Abs(+), immunoprecipitation assays	No	No	270 days
Mahmood et al [16]	2021	71	Male	Pembrolizumab 200 mg/Q3W	450 days	NSCLC	6.5 mg/dL (8.5-10.2 mg/dL), 1.62 mmol/L (2.1-2.5 pmol/L)	4.3 pg/mL (14-72 pg/mL), 0.45 pmol/L (1.5-7.6 pmol/L)	ND	No	No	420 days
Kreze et al [17]	2022	70	Male	Durvalumab 1500 mg/Q3W maintenance after chemo-RT	58 days	NSCLC	5.7 mg/dL (8.4-10.2 mg/dL), 1.42 mmol/L (2-2.55 mmol/L)	Undetectable (14.8-56.8 pg/mL, 1.57-6.03 pmol/L)	Anti-PTH Abs(−), ND	No	ND	ND
Present case	2024	65	Female	Ipilimumab (3 mg/kg), Nivolumab (1 mg/kg)	28 days	Metastatic Melanoma	6.3 mg/dL (8.5-10.5 mg/dL), 1.53 mmol/L (2.1-2.6 mmol/L)	8 pg/mL (14-65 pg/mL), 0.84 pmol/L (1.49-6.89 pmol/L)	Anti-PTH Abs(−), (indirect fluorescence immunoassay)	No	Yes	180 days

Abbreviations: Abs, antibodies; AUC, area under the curve; Ca, corrected calcium level; CaSR, calcium-sensing receptor; d, days; ELISA, enzyme-linked immunosorbent assay; ICI, immune checkpoint inhibitor; NALP5, NACHT leucine-rich-repeat protein 5; ND, no data; NSCLC, non-small cell lung cancer; PTH, parathyroid hormone level; Q3W: every 3 weeks; RT, radiotherapy; SCLC, small cell lung cancer.

It remains unclear whether ICIs have a direct impact on B-cell and antibody changes or indirectly cause a systemic dysregulation of immune self-tolerance or both [21, 22]. More specifically, the diversification and expansion of autoreactive CD4^+^ T-cells generated during immunotherapy may escape from the mechanism of self-tolerance. CD8^+^ T-cells may also be activated against healthy tissues as a consequence of the diversification and expansion of autoreactive clones. The antigenic cross-reactivity to self-antigens shared by tumor and healthy tissues is another etiology for an overreactive immune response as well as the proliferation and activation of B-cells, resulting in the secretion of antibodies that mediate the cell cytotoxicity in target tissues. Thus, the autoinflammatory destruction of the parathyroid gland may potentially involve not only the detection of CaSR antibodies that inhibit PTH secretion but also a multiple-level immune-mediated response ending in increased T-cell activity against parathyroid tissue [12]. The use of these autoantibodies in routine clinical practice, including autoantibodies to CaSR [23], to type 1 interferon (detected in 95% of patients with APS-1) [24] and to NACHT leucine-rich-repeat protein 5 (NALP5) [25] is limited due to their cost and lack of availability. Not exclusively in our case, any previous use of steroids may affect the level of immune response and the measurements of specific antibodies. Seronegative antibody-mediated irAEs have also been described in other irAEs explained by the abnormal release of cytokines and chemokines, which decrease the function of Tregs and the systemic and organ-specific inflammation or by the direct off-target effect of ICIs on cells bearing the targeted checkpoint ligand, as in the case of hypophysitis after anti-CTLA-4 treatment [22]. Even though the expression of PD-1/PD-L1 or CTLA-4 is unknown in normal parathyroid tissue, PD-L1 expression was demonstrated by immunohistochemistry in 8 (30.8%) of 26 parathyroid carcinomas and in 18 (48.6%) of 37 parathyroid adenomas [26]. Therefore, the ir-hypoparathyroidism could be defined as a type of autoinflammatory hypoparathyroidism with no specific immunological biomarkers for its diagnosis. The utility of autoantibodies is not sufficiently supported. Many other parameters, including the patient's immune homeostasis, the administered ICI regimen, the gut microbiome, and the host genetic background, are also implicated in its occurrence [22, 27].

According to the recent European Society of Endocrinology (ESE) guidelines, ICI-induced hypoparathyroidism is treated similarly to primary hypoparathyroidism due to other causes [28]. The major goal is to correct symptomatic hypocalcemia and to avoid short- and long-term complications, especially cardiological such as prolongation of the QT interval. Oral calcium and active vitamin D supplementation is the standard recommended treatment, while in cases of acute severe symptoms, intravenous calcium gluconate may also be required. Recombinant PTH and high-dose use of glucocorticoids are not recommended. Patients on ICI-based regimens should be routinely monitored for endocrine irAEs, including fasting venous glycemia, natremia, calcium and PTH levels, thyroid stimulating hormone, free T4, cortisol, adrenocorticotropic hormone, and gonadotrophins levels [29].

In conclusion, ir-hypoparathyroidism is a rare, irreversible, and potentially life-threatening endocrinopathy associated with ICIs. Indeed, all identified patients developed persistent primary hypoparathyroidism and the measurements of PTH did not recover many months after the initial diagnosis. Cases with ir-hypoparathyroidism and severe hypocalcemia may continue their immunotherapy, as limited other therapeutic options are available [30]. The discontinuation of immunotherapy has been previously described, that could not reverse the persistence of hypoparathyroidism [11, 12]. Calcium and PTH levels should be monitored at baseline prior to ICIs initiation and during treatment in clinical suspicion. The underlying pathophysiology of ir-hypoparathyroidism does not necessarily involve the formation of specific antibodies. Care providers should be aware of the entire spectrum of irAEs to promptly recognize and treat ir-hypoparathyroidism.

## Learning Points

- ICI-induced hypoparathyroidism is a rare and potentially life-threatening endocrinopathy.
- Calcium and PTH levels should be monitored at baseline prior to ICIs initiation and during treatment in clinical suspicion.
- The underlying pathophysiology does not necessarily involve the formation of specific antibodies.
- The reversibility of parathyroid gland function after ICI-induced hypoparathyroidism has not been reported yet in the existing literature.

## Contributors

All authors made individual contributions to authorship. H.G. was involved in conceptualization and supervision. S.K. did the literature search and data curation, S.K., D.Z., and An.A. were involved in original draft preparation. An.A, D.Z., and Am.A. were involved in reviewing and editing. All authors reviewed and approved the final draft.

## Data Availability

Original data generated and analyzed for this case report are included in this published article.

## Abbreviations

APS-1: autoimmune polyglandular syndrome 1
CaSR: calcium-sensing receptor
CTLA-4: cytotoxic T-lymphocyte-associated protein 4
ICI: immune checkpoint inhibitor
Ir: immune related
irAE: immune-related adverse event
PD-1: programmed cell death protein 1
PD-L1: programmed cell death ligand
PTH: parathyroid hormone
T4: thyroxine
